# The Woman Physician

**Published:** 1911-07

**Authors:** Sophia Brunson

**Affiliations:** St. Matthews, S. C.


### The Woman Physician.

BY DR. SOPHIA BRUNSON, ST. MATTHEWS, S. C.

When we consider the course and conduct of man, present and past, we see that he is largely guided by custom. Sons follow without question in the footsteps of their sires. The usages of the passing generation are adopted by the coming one, not because of their adaptability to present conditions, but because of their prevalence. Man reluctantly consents to change the customs of his ancestors. Prescriptive institutions, religious, social and political, acquire a peculiar sacredness in the eyes of man which forbids investigation, and repels the rude touch of the unsympathetic. Custom is the idol of society, and he who essays to change it is regarded as an iconoclast, a dangerous innovator worthy of the curse of the gods.

This unwise conservatism in man often leads to the retention for ages of customs that are positively harmful, and that impede the progress of the race. Witness, for example, in India the burning of widows upon the funeral pyres of their husbands, a practice which, despite its revolting cruelty, obtained for centuries, and which was reluctantly abandoned only in compliance with the peremptory command of the British government. Witness in China the binding of the feet of young girls, a performance which not only causes indescribable agony to the child, while a child, but which seriously impairs her efficiency as a woman. Witness in America the restricting of the waists of women, a fashion which destroys the natural symmetry and beauty of their figures, displaces the vital organs of their bodies, and unfits thousands for the sacred responsibilities of wifehood and maternity. Witness also the professional and political disabilities that have been imposed upon woman since the beginning of the human race, thereby depriving her of privileges that are justly her own and restricting her actions to a certain prescribed sphere. No good reason can be assigned for such prevailing practices as these. They are all in violation of natural laws, and are foolish, injurious and unjust. Yet in the face of the folly, injury and injustice they continue simply because they are custom. Our fathers did this and so do we.

Now progress demands the careful scrutiny of all inherited customs, and if necessary the judicious elimination of all those that are arbitrary. For permanent racial improvement is attained not by unquestioning compliance with arbitrary requirements but by intelligent obedience to laws that are in harmony with the nature and constitution of man. Whatever custom is contrary to nature, however old and venerable it may be, is an impediment to progress, because it restricts normal development.

Having said this much by way of introduction, we now approach the subject under consideration, viz.: Woman's place in medicine. Woman has entered the medical profession, and entered it to stay, though an inveterate conservatism, obstinate and unreasonable, has opposed her at every step. And this conservatism is not dead yet. True, it is in the throes of death, but it is dying hard. Wherever it can it flings its decrepit form athwart the highway of progress, and with a voice reminiscent of the plenary power which it once possessed, it says to the advancing hosts of women, "Stay. You are forbidden to enter here. This profession from time immemorial has been reserved for men, and we can not at this late day depart from the ways of our fathers. Stay. Stay. We can not admit you."

The experience of Madam Curie, the "heroine of radium," aptly illustrates the unreasonableness of the conservatism of which we have just been speaking. The Academy of Sciences refused to admit her to membership. Madam Curie happened to be born a woman, therefore conservatism says that she must not be admitted to membership in the Academy, however great her learning or valuable her services that she has rendered science. The question is one of sex, and not of scientific attainment.

But as we have just said, woman will not be baffled by any such exhibitions of fogyism. She has heard the call of humanity and she has risen to respond. She reasons correctly that her sex is not an adequate barrier to the enjoyment of privileges and opportunities to which she is entitled by natural equipment. The ancient custom that excludes her from the professions and restricts her wholly to the domestic sphere, simply because she is a woman, is unreasonable and therefore unjust. It is one of those arbitrary restrictions imposed by the will of man which hinders rather than helps the progress of the race, and the sooner it is abandoned the better. Woman is entitled to all the rights and privileges which man enjoys. Her field of action should be commensurate with her gifts. Endowment, not sex, indicates sphere. When her talents are great, and her ambition and taste, but more often the necessity of earning a livelihood for herself and others dependent upon her, lead her away from the quiet seclusion of home into the public arena, why should the fact that she is a woman restrict her? Why should it be considered indelicate for her to study the human body, that most wonderful and intricate piece of mechanism, and discover the function of each organ and the part it plays in the maintenance of health and efficiency? If she has the capacity and inclination for such vocations, that have been considered purely masculine, why should her sex prove to be a barrier?

Now in reasoning thus we do not mean to imply that woman should forsake the domestic sphere for which she is pre-eminently fitted and over which she has long presided. Not at all. The sacred duties and responsibilities of wifehood and maternity can not be neglected by woman without irreparable loss to mankind. The home is the cradle of the nation. Its influence is formative and directive. As are the homes so is the community. As are the communities so is the State. As are the States so is the nation. Home influence is all pervading. It develops character; character creates sentiment; sentiment moulds public opinion; public opinion shapes the policy of the nation. Thus society in the broadest and most comprehensive sense of the term, is but a reflex of the home; and woman is the home-maker.

For this delicate task which we all recognize to be of prime importance, woman is peculiarly fitted by nature. A true womanly woman who, with unselfish soul and lofty purpose, so controls and directs the affairs of a home as to create an atmosphere of wise contentment and peace, and so moulds the plastic souls of nascent youths as to guide their expanding minds into channels of pure and worthy thought, is doing a work that is second to none in the world. And from this noble work which is fundamental to all others, nobody in his right mind would for a moment attempt to dissuade woman. In this she occupies a sphere that is peculiarly her own, a sphere which no other can fill. Here she has wrought her greatest work and has proved herself to be a tremendously potent factor in the development of the human race.

But when all this has been said still we plead for the right of woman to enter the professions if she chooses. As a matter of fact most women prefer the quietude of home, and it is well that they do. But there are many who do not, and again others who are compelled to support themselves. They are not suited by nature for housekeepers, teachers nor dressmakers. Their talents and tastes lead them to desire different fields of activity. Some prefer law, some dentistry, some medicine, and so on. They are endowed by nature with capacity that promises success. Then when a woman finds herself thus endowed, should she be denied the privilege of following the bent of her mind simply because she is a woman? She had no voice in the creating and equipping of herself. She did not bestow upon herself the mental qualities which lead her to seek a sphere of action in the more public walks of life. That was the work of her Creator, and it indicates his purpose in equipping her. We repeat that endowment points to sphere, and when the Creator gives to a particular woman a particular endowment which fits her for successful work in a particular profession, and her taste and ambition incline her to choose that profession, why should her sex stand in her way? It seems to us that under such conditions a sense of fairness and justice unequivocally decrees that she should be allowed the same freedom of choice that is granted man. Sex should not be considered.

And now we come to speak more particularly in regard to the medical profession. Here is a sphere for which woman is peculiarly adapted and in which she is capable of rendering most efficient service. The woman physician is needed. There is a distinct work for her to do, and there ought to be one or more in every community. In speaking thus we are not disparaging the work of the male physician, nor implying that he should be supplanted by the female. But when we consider the large number of women and girls who need medical attention and who are restrained by modesty from submitting to examinations by male physicians, and the great amount of suffering and the large number of premature deaths that are indirectly caused thereby, we do asseverate that the place of the competent female physician can not be successfully filled by one of the opposite sex. Every woman physician of large experience could, if necessary, tell of scores of cases of delayed recovery, of prolonged suffering, of premature senility, all because the patient was a timid, shrinking woman and her physician a man. It is needless to say that such modesty on the part of woman is foolish, and that when health and efficiency is involved it ought to be laid aside. Foolish or wise, it exists.

But not only is the woman physician needed in general practice and in hospitals, but there ought to be one in every college for women. This is a crying necessity that has been too long neglected. If the patrons of our female colleges were alive to the needs and best interest of their daughters, they would peremptorily demand women physicians in these schools. Many girls leave college after a comparatively brief stay, nervous wrecks, and some are injured for life because they lacked the proper attention at the proper moment. The girl student leaves home for college at a critical period of life. She is just entering the age of puberty, and is beginning to become conscious of herself as a woman in contradistinction to man. The innocency and simplicity of childhood are passed and the intricate problems of womanhood are beginning to appear. Her cerebral system is rapidly developing and her nerves are in a state of unstable equilibrium. A false step at this stage might prove permanently disastrous to her body, her mind or her character, or to all three. A mother's counsels are needed then if ever. And yet she is taken from home at that critical period and placed in a boarding school under the medical supervision of the college physician, a man and a stranger at that. Through some youthful indiscretion of one kind or another she perhaps is made sick, not serious at first. She neglects herself as the young will do, till finally some complication that is peculiar to females arises. There is no one to whom she can unburden herself, and often she gives up in discouragement and goes home to linger for months and years in a state of semi-invalidism. If there had been connected with the college a female physician to whom she could have gone and spoken freely, the error would have been corrected in its incipiency, and years of suffering avoided. Every school in which girls are taught should have a chair of physiology and practical hygiene occupied by a woman physician. And the girls ought to be taught to care for themselves properly, and instructed in regard to the physiological functions of the vital organs of the body. Instruction in dress, diet, exercise, breathing, sleeping, eating—all should be included in the college curriculum. Moreover, since every normal girl is a prospective wife and mother, she ought to be taught in regard to the care of children. Such instruction is practical, and if received by the majority of our women would result in the permanent uplift of the race.

So we repeat in conclusion that the woman doctor has come to stay. She is needed and will be as long as there are ailing women and suffering girls. She seems to be especially fitted by nature for the work to which she devotes herself. She possesses tenderness and tact, kindness and considerateness, sympathy and suavity, and when in the sick room the gentleness of her manners, the softness of her voice, and the delicateness of her touch, all tend to soothe the sufferer and to allay the irritation of his nerves. She easily wins the confidence of her patients and binds them to herself by strong and enduring ties of affection. In a word her presence is a boon to the women of any community because she meets a long felt want.

And yet there are old fogies who are raising the cry that it is indelicate for women to study medicine. That she is out of her place. Why is it that we never hear anything about the work of the trained nurse being indelicate and unwomanly? It is because the trained nurse works under the directions of the male physician and is subservient to him, therefore her work is considered perfectly proper, no matter how menial it may be, nor how much the persons of her male patients are exposed in her presence. Still it is considered very improper by some people for the two sexes to study side by side in the same medical college. "To the pure all things are pure." How can there be anything immodest for men and women of mature minds to study together the great truths that pertain to the human body, especially when the object in view is such a lofty one, viz., to uplift mankind, to heal their diseases, and to teach them how to live? Common sense and true modesty can see nothing improper in such association of the two sexes. Only the false and prurient thing that we call prudery, but more properly the jaundiced eye of prejudice, can detect anything indelicate in such intercourse.

The treatment accorded Elizabeth Blackwell, that great pioneer woman physician, who established in 1853 "The New York Infirmary for Women and Children," and which proved so successful that in 1868 she founded the "Woman's Medical College," which was afterwards converted into Cornell University, is an illustration. When this noble hearted woman who did so much for humanity went abroad that she might increase her knowledge and augment her usefulness by studying in the colleges of Europe, she was told in Paris that it would be impossible for her to gain entrance to the schools or hospitals there unless she adopted male attire. That was long ago, and yet the College of Charleston is shutting its doors to women, not because they lack the brain or capacity to study medicine, but apparently only because they are women. Is it right that our girls who wish to be educated in the professions, or along advanced lines the better to serve humanity, should be compelled to go North to receive such training? No, a thousand times, no! Where is our boasted Southern chivalry? Where is our sense of right and justice? Our girls are only asking the right to be trained and to develop their God-given talents in this their own loved, native Southland.

We can not stem the tide of progress by our puerile prejudices and conventions; for the time is coming when our girls will not have to stand and knock upon closed doors and beg for opportunities for the training accorded to their brothers but denied to them. And enlightment and right at last will triumph, and prejudice and injustice will hide their heads in shame, and die as they deserve, even as do the germs of disease in the poisons which they manufacture.—*Charlotte Medical Journal.*

